# Mode Effects Between Telephone and Web Interviews in the Post-COVID-19 Questionnaire Survey CoVerlauf: Exploratory Study

**DOI:** 10.2196/80631

**Published:** 2026-03-06

**Authors:** Paula Sofia Herrera-Espejel, Hermann Pohlabeln, Lisa Kühne, Stefan Rach

**Affiliations:** 1 Department of Epidemiological Methods and Etiological Research, Leibniz Institute for Prevention Research and Epidemiology - BIPS, Achterstr. 30, Bremen, 28359, Germany, 49 (0)42121856 ext 841; 2 Leibniz ScienceCampus Digital Public Health, Bremen, Germany; 3 Department of Statistical Methods in Epidemiology, Leibniz Institute for Prevention Research and Epidemiology - BIPS, Bremen, Germany; 4 SOCIUM Research Center on Inequality and Social Policy, Department of Health, Long-Term Care and Pensions, University of Bremen, Bremen, Germany

**Keywords:** concurrent mixed mode, data collection, CATI, CAWI, population-based, CoVerlauf, post-COVID-19, self-selection effect, mode measurement effect

## Abstract

**Background:**

Concurrent mixed mode designs for data collection may introduce mode-specific biases. This study investigated mode effects between computer-assisted website interviewing (CAWI) and computer-assisted telephone interviewing (CATI) in the concurrent mixed mode study CoVerlauf.

**Objective:**

This secondary analysis of previously published results explored associations between the choice of interview mode and socioeconomic characteristics, the number of reported post-COVID-19 symptoms, and response patterns in symptoms collected via multiple-choice (MC) and free-text items.

**Methods:**

Associations were calculated using multiple logistic regression analyses, mixed effects logistic regression analyses, and gamma generalized linear models. Selection and measurement effects in symptom counts were analyzed with the back-door method.

**Results:**

Among 1779 participants, 77.8% (1384/1779) chose CAWI, and 22.2% (395/1779) chose CATI. Odds of selecting CATI were lower with a higher education (odds ratio [OR] 0.46, 95% CI 0.34 to 0.63), for those represented by another person (OR 0.28, 95% CI 0.13 to 0.56), and with each additional symptom experienced at the time of the interview collected from MC (OR 0.86, 95% CI 0.77 to 0.96). Compared with those aged 50 years to 59 years, the odds increased with age (60‐69 years: OR 2.26, 95% CI 1.53 to 3.36; 70‐79 years: OR 4.98, 95% CI 3.12 to 7.97; ≥80 years: OR 17.29, 95% CI 9.34 to 33.11) and each additional symptom experienced at infection collected via MC (OR 1.09, 95% CI 1.04 to 1.15) and free-text items (OR 1.94, 95% CI 1.61 to 2.37). Odds of responding to free-text items were higher with CATI (OR 5.22, 95% CI 4.25 to 6.42), for women (OR 1.57, 95% CI 1.30 to 1.89), for those with ≥3 pre-existing conditions (OR 1.44, 95% CI 1.07 to 1.96), with missing information on educational attainment (OR 1.95, 95% CI 1.11 to 3.42), and for each additional MC symptom (OR 1.15, 95% CI 1.12 to 1.19). Odds of collecting additional symptoms from free-text items were lower with CATI (OR 0.58, 95% CI 0.41 to 0.82) but increased with each additional MC symptom (OR 1.08, 95% CI 1.02 to 1.14), ≥2 pre-existing conditions (2: OR 1.8, 95% CI 1.02 to 3.00; ≥3: OR 2.0 95% CI 1.18 to 3.43), and ages 40 years to 49 years (OR 2.0, 95% CI 1.13 to 3.55). Selection effects were found for the number of symptoms at infection collected via MC (difference –0.63, 95% CI –0.95 to –0.31) as were measurement effects for symptoms collected via MC at infection (difference 0.97, 95% CI 0.47 to 1.5), via free text at infection (difference 0.40, 95% CI 0.29 to 0.52), and via free text at the time of the interview (difference 0.15, 95% CI 0.05 to 0.25).

**Conclusions:**

Our results suggest that interviews via CATI resulted in higher numbers of reported symptoms collected via MC and free text and that both selection and measurement effects contributed to this increase. However, no evidence was found that the interview mode affected the overall findings of the CoVerlauf study.

## Introduction

### Background

Researchers often offer study participants the choice among different interview modes (eg, online, telephone, in person, or paper and pencil) with the purpose of broadening participation [1]. Computer-assisted website interviewing (CAWI) and computer-assisted telephone interviewing (CATI) are two modes commonly used as data collection methods in population-based studies. CAWI is a self-administered, web-based reporting method in which respondents are provided with a link to an online questionnaire that they generally complete on their own and on an internet-enabled device of their preference [2]. CATI, by contrast, is an interviewer-administered method, where trained interviewers serve as intermediaries to ask each question aloud and record responses using a combination of telephone communication and computer-guided technology [3]. Although CAWI mainly uses visual communication channels handled by the participants themselves, CATI uses aural communication and relies on the presence of the interviewers to capture responses.

The use of different interview modes can influence how study data are collected. On the one hand, preferences for interview modes can be significantly associated with selection effects: Participants picking the same mode may also share similar socioeconomic and demographic characteristics, including their use of digital technology [4]. For example, younger age groups from higher socioeconomic backgrounds and urban areas might be more inclined to use digital communication methods, while older age groups or those who habitually stay at home (eg, due to unemployment) might prefer analog ones [1] for exchanging information [5-8]. On the other hand, the use of different interview modes may also lead to measurement effects: systematic variations in how participants respond to identical questions caused by mode-specific intrinsic features (see [9,10]). For instance, interview modes involving a direct interaction with interviewers are subject to different kinds and degrees of information bias than self-administered modes (eg, diagnostic bias or social desirability bias). Collectively, population-based studies may face a situation where participants with similar characteristics choose the same interview mode, which, in turn, subjects them to data collection procedures that induce systematically different response patterns.

Existing research on the effects of mode-specific differences between CAWI and CATI (eg, visual vs aural communication channels and absence vs presence of interviewer, respectively) on response frequency and content has yielded mixed results. From one side, research suggests that CAWI respondents may be more likely to complete online questionnaires with minimal time and cognitive effort, providing shorter, if not incomplete, answers than with other modes [11]. From another side, there is evidence that CAWI may lead to more sincere and extensive answers because it allows participants to respond more calmly, at one’s own pace, and with a higher sense of privacy [7]. Correspondingly, the odds of social desirability bias (ie, inclination to adjust answers to conform with anticipated expectations and norms) may vary between interview modes when interrogated about sensitive health topics. For instance, research reports lower odds of social desirability when using CAWI for answers concerning HIV testing [12], whereas higher odds are reported when using CATI for answers about symptoms of infectious diseases, mental health, or substance abuse due to the presence of an interviewer [13-15].

At the same time, research on interviewer effects has indicated that the nature of the spoken interaction in CATI results in a higher number of responses than in self-administered modes, such as CAWI [16]. Furthermore, related research shows mixed results on the effect of the different modes depending on whether the questions are with or without predefined response categories (ie, multiple-choice vs free-text items). Although one meta-analysis found that item response behavior was similar between question formats across all methods [17], a more recent study suggested that CATI respondents systematically selected more options within multiple-choice items, resulting in higher item response proportions to certain questions than with CAWI [18]. Similarly, another study concluded that CAWI questionnaires resulted in fewer free-text items overall compared with CATI [11].

Aside from frequency of response, another line of research has also pointed to between-mode differences in the content of answers. For example, the content of free-text items regarding satisfaction with particular topics in CAWI may be more likely to contain negative feedback than that in CATI [19]. Past studies have also demonstrated that free-text items are particularly prone to interviewer and recall effects in spoken interactions, potentially resulting in differential reporting between CATI and CAWI [16,20,21]. The literature also suggests that, although CAWI might induce primacy effects (eg, inclination to select the first options read from a list of options) [22,23], it is unclear whether CATI is more susceptible to either primacy or recency effects (eg, inclination to select the last options presented from the same list read aloud) [13].

These findings are reiterated in medical and epidemiological literature specifically studying the effect of interviewer- and self-administered mode choices and question format on response patterns. For example, Milton and colleagues [24] found that, related to sensitive mental health and well-being topics, the reporting of symptoms by young Australians (aged 16 years to 25 years) is dependent on whether the interview is self-administered. Overall, they observed a higher disclosure of such high-sensitive items in online-based surveys. Similarly, Zager Kocjan and colleagues [25] highlighted that merging data collected from items asking on psychological functioning from different modes into a single dataset may result in structural noninvariance with significant mean differences between these two types of modes. Namely, the authors observed a greater tendency for respondents to report fewer depressive symptoms when interviewers were directly involved in data collection, potentially resulting in the underestimation of the actual prevalence or severity of symptoms compared with responses collected anonymously through an online portal. The auditory versus visual nature of the interview is also found to have systematic differential response order effects on how respondents report sensitive symptoms [26]. For example, Cernat and colleagues [26] found that items collected from the Center for Epidemiologic Studies Depression scale are sensitive to primacy and recency effects depending on the interview mode. Although CAWI respondents tended to select items from the beginning of the lists, CATI respondents were more likely to choose those at the end of the list.

### Research Objective

This study investigated the potential effects of using different interviewing modes (CATI vs CAWI) on participant response patterns to questions about symptoms experienced in the CoVerlauf study. Conducted in Germany in 2021, the CoVerlauf study provided evidence on the odds of experiencing a post-COVID-19 condition after contracting a verified SARS-CoV-2 infection [27]. The study concluded that more than 12% of participants (203/1612, 12.6%) who reported at least one symptom at the time of their infection experienced symptoms of a post-COVID-19 condition 3 months after the infection (ie, ongoing symptoms of fatigue, breathing difficulties, and cognitive complaints following their initial infection). Since all enrolled participants could select either CAWI or CATI to complete the study questionnaire, mode effects might have impacted data collection and findings. In particular, the choice of the interviewing mode may have not only been associated with the participants’ characteristics and health status but also influenced how participants interacted with the multiple-choice and free-text items beyond their shared characteristics.

This study serves two main research objectives: (1) to determine whether there is an established link between the participants’ choice of the interview mode (CAWI vs CATI); their health, socioeconomic, and demographic characteristics; and the number of reported post-COVID-19 symptoms; and (2) to identify whether the free-text items regarding if participants experienced “any other symptom” aside from those listed in the multiple-choice items systematically differed between the two mode groups.

## Methods

### Ethical Considerations

All procedures in the CoVerlauf study were performed in accordance with the ethical standards of the institutional or national research committee and with the 1964 Helsinki declaration and its later amendments or comparable ethical standards. The CoVerlauf study was approved by the Ethics committee of Bremen University (reference number 2020‐05-EILV). Written consent was obtained from all participants in the study or the legal guardians of those aged younger than 18 years. Participation was voluntary and without any monetary or nonmonetary compensation. The article does not include personal information, identifying details, or identifiable features of research participants.

### Study Design

This study used existing data from 1779 individuals with a verified SARS-CoV-2 infection aged between 1 year and 98 years from the City Municipality of Bremen, Germany, who participated in the CoVerlauf study [27]. A detailed description of the study design and the recruitment protocol as well as the questionnaire in German and in English can be found in [27]. Briefly summarized, CoVerlauf explored how even mild cases of COVID-19 could also be associated with a post-COVID-19 condition, defined as reporting at the time of the participants’ interview 3 months after the infection at least two of the following symptoms: fatigue, a cognitive disorder, or breathing difficulties. The study used a questionnaire survey to collect self-reported participant data on the symptoms they experienced at 2 different time points. Family members or caretakers could also answer the questionnaire in the place of persons unable to answer the questionnaire by themselves (eg, minors, older adults, or incapacitated persons). Persons were invited via land mail by the Public Health Department of the City Municipality of Bremen (Germany) if they had either tested positive for SARS-CoV-2 or been diagnosed with COVID-19 by a physician between March 1, 2020, and January 31, 2021. One reminder letter was sent out 12 weeks to 16 weeks after the initial invitation letter. Enrolled participants were first contacted by field staff to determine their preferred interviewing mode for completing the questionnaires (CATI or CAWI; concurrent mixed mode design [28]). CATI interviews were conducted by study nurses who all had multiple years of experience in recruitment and data collection for epidemiologic population-based studies and were trained in conducting standardized interviews. To record participants’ answers, interviewers used a special version of the online questionnaire that, in addition to the questions, included instructions and transitional statements to be read out during the interviews [2]. CAWI questionnaires were self-administered by the participants.

A first set of questions asked participants to recall the symptoms they experienced at the time of their infection and the first weeks afterward (hereafter, referred to as “time of infection”). A second set of questions asked participants about their long-term symptoms at the time of the interview, which was conducted at least 90 days after their diagnosis (hereafter, “time of interview”). Two question formats were used to collect the information on symptoms experienced at both time points. First, participants were provided with a multiple-choice list of 13 common COVID-19 infection symptoms (common at the time of questionnaire inception): fatigue, respiratory problems, diarrhea, fever, joint pain, scratchy throat, cough, headache, sniffles, chills, nausea, loss of taste, and loss of smell. Second, a free-text item offered the opportunity to provide additional symptoms.

Free-text items were subjected to a word frequency analysis that resulted in the classification of 15 additional symptoms: “hair loss,” “tightness of chest,” “circulatory problems,” “cognitive symptoms,” “dermatological skin symptoms,” “eye symptoms,” “gastrointestinal symptoms,” “heart problems,” “loss of appetite,” “mental difficulties,” “nose & ear symptoms,” “sensory skin symptoms,” “sleep difficulties,” and “flushing & sweats.” The free-text items were then subject to an independent screening, coding, and consensus procedure by 2 reviewers. The study also gathered data on the participants’ weight and height, pre-existing conditions, and socioeconomic status. Additionally, the duration required to complete the questionnaire was automatically logged by the CAWI and CATI software and measured in minutes for each respondent. In 4 instances, the interview duration was longer than 1000 minutes, indicating that the interview was interrupted for more than 16 hours (CATI: 3; CAWI: 1). These outliers were excluded for the creation of figures (maximum duration after removal: 110 min) and replaced with imputed values using linear regression imputation with the MICE (multivariate imputation by chained equations) algorithm [29] for the regression analysis. A sensitivity analysis, which involved repeating the regression as a complete-case analysis excluding the 4 outliers, revealed no differences (Multimedia Appendix 1).

### Statistical Methods

This study first examined the interviewing mode preference (CATI vs CAWI) of participants to the CoVerlauf study. A multiple logistic regression analysis was conducted to assess the relationship between the choice of mode and the number of symptoms collected from answers to the multiple-choice and the free-text items at the time of infection and interview separately. The corresponding regression model (equation 1 in Multimedia Appendix 2) included sociodemographic and health indicators as explanatory variables, namely sex (female vs male), age categorized into 8 age groups (0‐17, 18‐29, 30‐39, 40‐49, 50‐59, 60‐69, 70‐79, ≥80 years), educational attainment based on the International Standard Classification of Education (ISCED) scale (low: 1, 2; medium: 3, 4; high: 5, 6), weight status categorized according to BMI and calculated from self-reported body weight and height (normal: BMI <25 kg/m²; overweight: BMI 25‐30 kg/m²; obese: BMI ≥30 kg/m²), and the number of pre-existing conditions (0, 1, 2, vs ≥3). The model also included a dichotomous variable “self-reported” (“self” vs “proxy”) to differentiate whether the participant self-reported their answers or had another person answer on their behalf. As in the original publication [27], missing values in the variables for weight status and educational attainment were coded as a separate category “Missing.”

Furthermore, the study investigated the role of the mode in response patterns to the 2 free-text items on additional symptoms through 2 separate mixed effects multiple logistic regression analyses (equations 2 and 3 in Multimedia Appendix 2). For theses analyses, data were restructured into the long format, where participants were represented with 2 rows: one for their responses at the time of infection and another for those at the time of interview. As such, the 2 models were conducted on a dataset with twice the number of observations than the number of participants considered in each of the analyses. The first model examined the association between the mode (CATI or CAWI) and answering free-text items (“response” vs “no response”). The second model focused on the subset of individuals who answered these questions, assessing how the interview mode influenced the probability of identifying and collecting at least one or more additional symptoms (+1 symptom) compared with none (0 symptoms). Both analyses included the aforementioned sociodemographic and health variables. To account for potential dependencies within individuals, both models incorporated random effects for responses from the same individuals (ie, ID) and time point of the reported symptoms (ie, time of infection and time of interview).

A gamma generalized linear regression model with log link function was used to analyze the outcome variable of overall interview duration (in minutes) and estimate time differences between the 2 mode groups (equation 4 in Multimedia Appendix 2). The choice of the model was based on the overall right-skewed distribution and because the outcome variable is continuous.

The back-door method was used to disentangle selection and measurement effects in the observed mode effects on symptom counts [30,31]. Derived from causal inference theory, this method consists of comparing answers to one of the interview modes (principal mode) to counterfactual answers generated from other modes (alternative modes) that assume the respondents had used the principal mode. To create counterfactual answers, propensity score weights are calculated from a set of variables that are assumed to be insensitive to the choice of the interview mode (eg, age). Outcome differences between the unweighted and weighted answers in the alternative mode are assumed to capture selection effects, whereas differences between the weighted alternative mode and the principal mode quantify measurement effects. For the current analysis, CAWI was selected as the principal mode, and a set of counterfactual answers CATI_W_ was created using propensity score weights, which were calculated using the interview mode as the outcome variable and sex, age category, educational attainment (low and medium vs high), and number of pre-existing conditions (0, 1, and 2 vs ≥3) as independent variables. Mean differences and 95% CIs were calculated for each of the 4 symptom counts (ie, from the multiple-choice and free-text items at time of infection and interview). In addition, we calculated standardized mean differences (SMDs) with 95% CIs to make the effects comparable across outcome variables.

All statistical analyses were executed using R version 4.3.3. The logistic regression model was executed using the stats package [32], while the mixed effect logistic regression models used the lme4 package in R [33]. Propensity score weights were calculated using the package WeightIt [34], and SMDs were calculated using the package smd [35]. Results for all regression models with interview mode as outcome are reported as odds ratios (OR) with 95% CIs, and results from the model with duration as outcome are reported as mean ratios (MRs) with 95% CIs. Subgroup differences were assessed with Pearson *χ*^2^ tests with Yates continuity correction.

## Results

**Figure 1.**
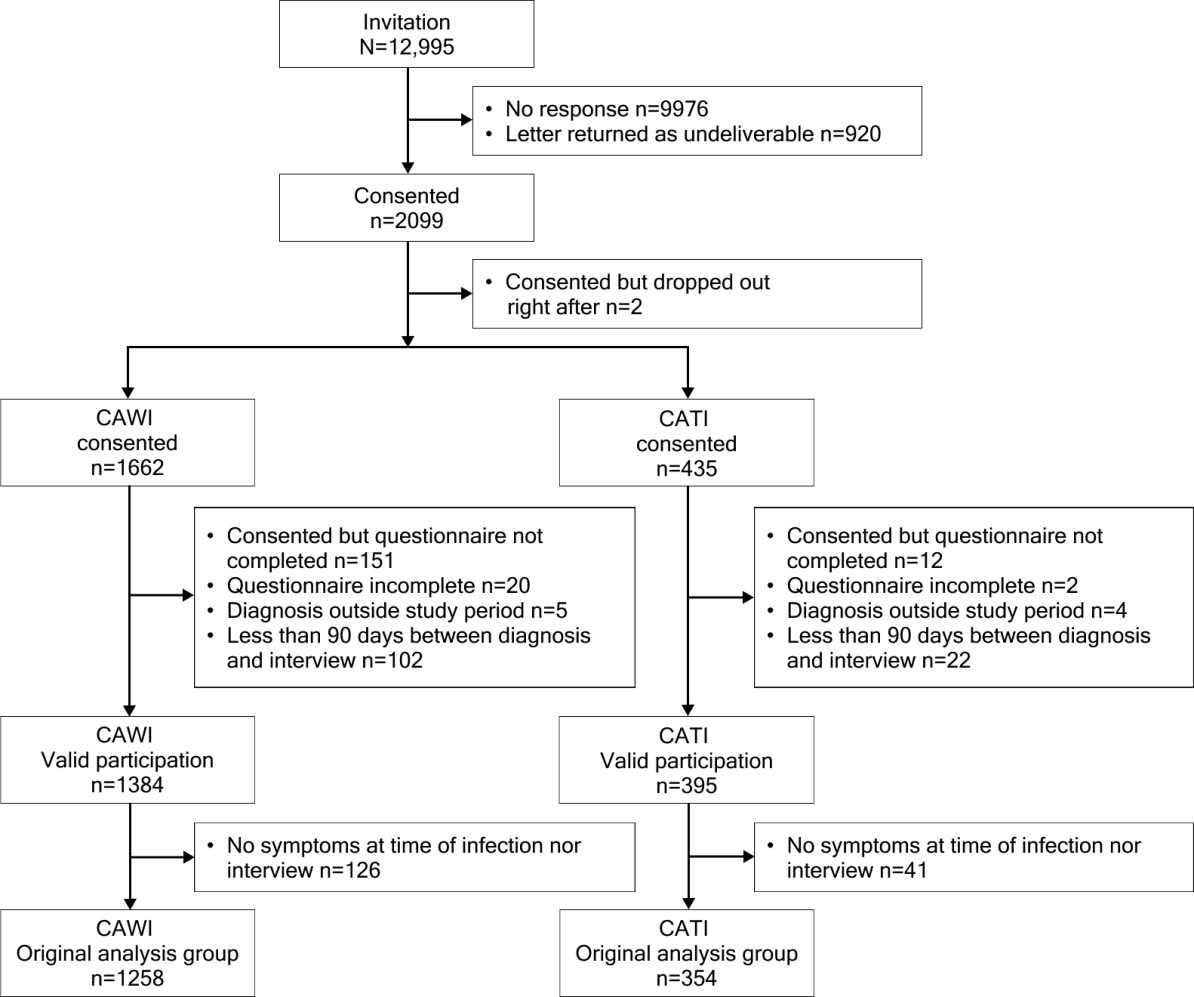
Flowchart of study participants across interview modes of computer-assisted website interviewing (CAWI) and computer-assisted telephone interviewing (CATI).

Among all enrolled participants (n=1779), 77.8% (1384/1779) used CAWI, and 22.2% (395/1779) used CATI to complete the CoVerlauf questionnaire (Figure 1).

**Figure 2.**
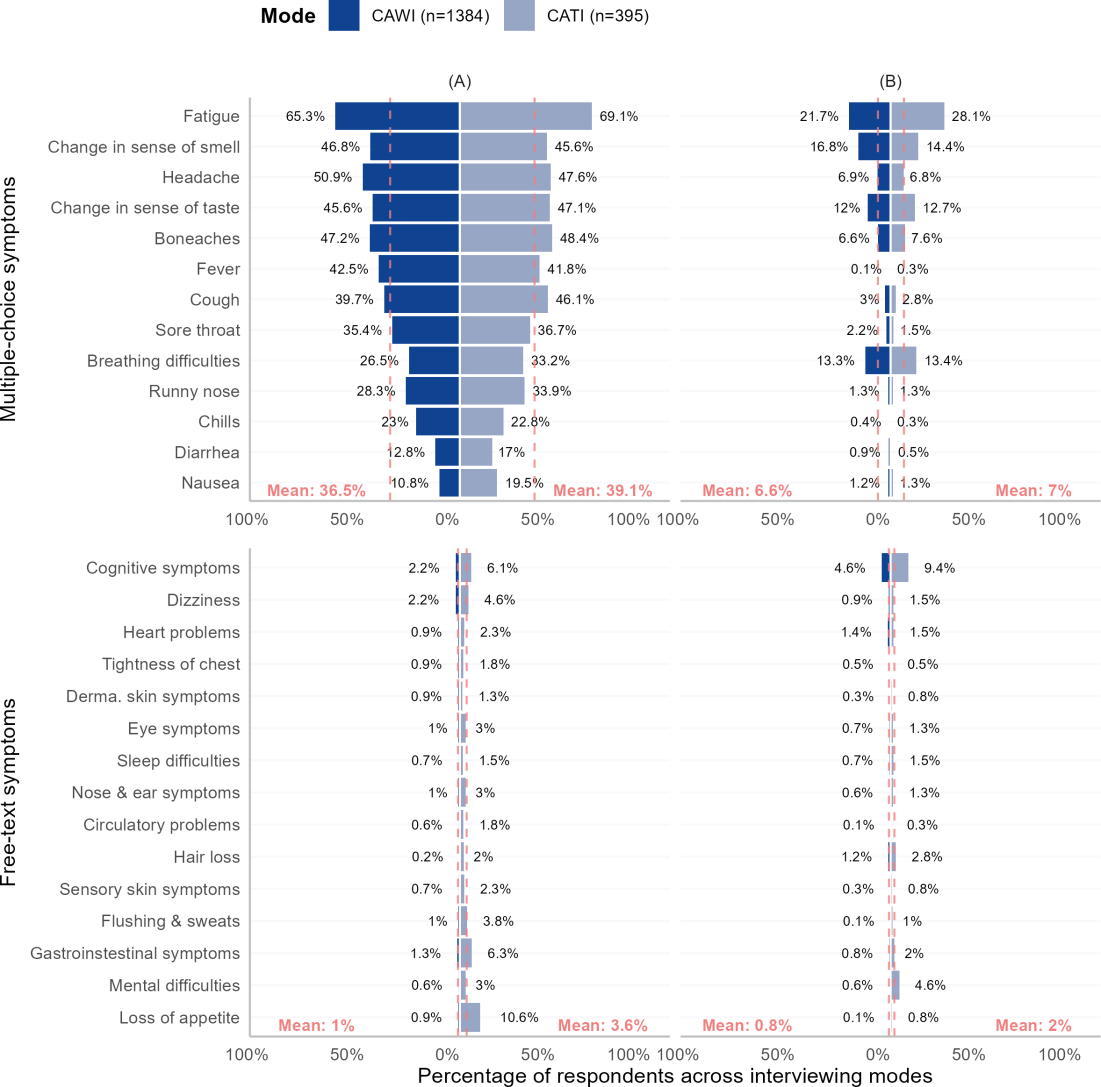
Comparison of percentage of participants reporting post-COVID-19 symptoms at the (A) time of infection and (B) time of interview in multiple-choice and free-text items across interviewing modes. CATI: computer-assisted telephone interviewing; CAWI: computer-assisted website interviewing.

Compared with CAWI participants, the percentages of participants reporting symptoms was consistently higher with CATI at both time points and across the 13 multiple-choice and 15 free-text items (Figure 2).

The multiple logistic regression analysis assessing associations between sample characteristics and the choice of CATI against CAWI revealed significant differences across most age groups, educational ISCED attainment categories, and the number of symptoms submitted at both the time of infection and interview for both question formats (Table 1). The odds of choosing CATI were lower for participants with a high education (OR 0.46, 95% CI 0.34‐0.63) relative to those with an upper-secondary education level. In comparison with participants 50 years to 59 years old, participants became more likely to choose CATI with increasing age (60‐69 years: OR 2.26, 95% CI 1.53‐3.36; 70‐79 years: OR 4.98, 95% CI 3.12‐7.97; ≥80 years: OR 17.29, 95% CI 9.34‐33.11). Those in age groups younger than 50 years to 59 years old were increasingly less likely to choose CATI, although ORs significantly differing from 1 were only observed in the age group 18 years to 29 years (OR 0.56, 95% CI 0.33‐0.93). Participants who had their questionnaires completed by another person (ie, proxy respondents) were overall less likely to use CATI (OR 0.28, 95% CI 0.13‐0.56) than those who responded for themselves. Note that participants in the lowest age group were 1.6 times more likely to use CATI (OR 1.63, CI 0.80‐3.32), which might be partly explained by the high proportion of their questionnaires completed by a proxy respondent (Multimedia Appendix 3).

**Table 1.** Sample characteristics by interview mode and odds ratios for selecting computer-assisted telephone interviewing (CATI) over computer-assisted website interviewing (CAWI) from multiple logistic regression analysis.

Predictors	Interview mode (n=1779), n (%)		Odds ratio (95% CI)	
CATI (n=395)	CAWI (n=1384)		
N_Sx_^a^ infection MC^b^	5.09 (3.40)^c^	4.75 (2.93)^c^	1.09 (1.04‐1.15)	
N_Sx_ infection free-text^d^	0.534 (0.899)^c^	0.150 (0.540)^c^	1.94 (1.61‐2.37)	
N_Sx_ interview MC	0.909 (1.26)^c^	0.862 (1.38)^c^	0.86 (0.77‐0.96)	
N_Sx_ interview free-text	0.299 (0.745)^c^	0.126 (0.443)^c^	1.21 (0.94‐1.54)	
Sex				
Male	170 (43)	590 (42.6)	1 (reference)	
Female	225 (57)	794 (57.4)	0.85 (0.65‐1.10)	
Age group (years)				
0‐17	25 (6.3)	102 (7.4)	1.63 (0.80‐3.32)	
18‐29	26 (6.6)	211 (15.2)	0.56 (0.33‐0.93)	
30‐39	28 (7.1)	222 (16)	0.72 (0.43‐1.19)	
40‐49	39 (9.9)	234 (16.9)	0.89 (0.57‐1.39)	
50‐59	76 (19.2)	339 (24.5)	1 (reference)	
60‐69	78 (19.7)	181 (13.1)	2.26 (1.53‐3.36)	
70‐79	62 (15.7)	73 (5.3)	4.98 (3.12‐7.97)	
≥80	61 (15.4)	22 (1.6)	17.29 (9.34‐33.11)	
Weight status according to BMI categories				
Normal	183 (46.3)	698 (50.4)	1 (reference)	
Overweight	116 (29.4)	437 (31.6)	0.81 (0.59‐1.11)	
Obese	95 (24.1)	242 (17.5)	1.10 (0.76‐1.57)	
Missing	1 (0.3)	7 (0.5)	0.58 (0.03‐3.52)	
Number of pre-existing conditions				
0	146 (37)	784 (56.6)	1 (reference)	
1	110 (27.8)	310 (22.4)	1.24 (0.89‐1.72)	
2	64 (16.2)	156 (11.3)	1.21 (0.80‐1.83)	
≥3	75 (19)	134 (9.7)	1.06 (0.68‐1.63)	
Education (ISCED^e^)				
Low (1, 2)	63 (15.9)	155 (11.2)	1.48 (0.92‐2.35)	
Medium (3, 4)	238 (60.3)	635 (45.9)	1 (reference)	
High (5, 6)	87 (22)	543 (39.2)	0.46 (0.34‐0.63)	
Missing	7 (1.8)	51 (3.7)	0.46 (0.16‐1.14)	
Proxy versus self				
Self	381 (96.5)	1307 (94.4)	1 (reference)	
Proxy	14 (3.5)	77 (5.6)	0.28 (0.13‐0.56)	

a N_Sx_: number of symptoms.

b MC: multiple-choice item.

c Mean (SD).

d Free-text item.

e ISCED: International Standard Classification of Education.

The odds of selecting CATI increased by 9% and almost doubled for each additional symptom collected from the multiple-choice (OR 1.09, 95% CI 1.04‐1.15) and free-text (OR 1.94, 95% CI 1.61‐2.37) items inquiring about symptoms experienced at the time of infection. The odds of selecting CATI were 14% lower for each additional symptom collected from the multiple-choice items inquiring about symptoms experienced at the time of interview (OR 0.86, 95% CI 0.77‐0.96). Although the 95% CI included 1, these odds increased for symptoms collected from the free-text items (OR 1.21, 95% CI 0.94‐1.54).

The odds of responding to the free-text items across time points (infection and interview) were calculated using mixed effects multiple logistic regression analysis (left columns in Table 2). Note that the unit of observations in this analysis is each of the 2 free-text items (n=3558) answered by each participant, rather than the participants themselves (n=1779). Results indicated that participants using CATI were nearly 5 times more likely to respond to the free-text items than those using CAWI (OR 5.22, 95% CI 4.25‐6.42). Female participants were 57% more likely to provide additional information (OR 1.57, 95% CI 1.30‐1.89). Participants who reported 3 or more pre-existing conditions were 44% more likely to answer the free-text item (OR 1.44, 95% CI 1.07‐1.96) than those reporting no pre-existing conditions. For each additional symptom collected from the multiple-choice items, the odds of responding to the free-text items increased by 15% (OR 1.15, 95% CI 1.12‐1.19). Participants in age groups younger than 39 years (0‐17 years: OR 0.46, 95% CI 0.25‐0.82; 18‐29 years: OR 0.63, 95% CI 0.45‐0.87; 30‐39 years: OR 0.59, 95% CI 0.42‐0.81) and those in the age group of 70‐79 years (OR 0.63, 95% CI 0.43‐0.91) were less likely to respond to the free-text items than those aged 50 years to 59 years. Participants with missing ISCED information were nearly twice as likely to respond to these items (OR 1.95, 95% CI 1.11‐3.42).

**Table 2.** Sample characteristics and odds ratios (ORs) calculated from mixed effects logistic regression models for responding to the free-text items and for collecting at least one additional post-COVID-19 symptom from the free-text items.

Predictors	Participant responded to free-text items (n=3558)				At least one additional symptom collected from free-text item content (n=729)			
	Yes (n=729), n (%)	No (n=2829), n (%)	OR (95% CI)		Yes (n=477), n (%)	No (n=252), n (%)	OR (95% CI)	
Mode								
CAWI^a^	374 (51.3)	2394 (84.6)	1 (reference)		265 (55.6)	109 (43.3)	1 (reference)	
CATI^b^	355 (48.7)	435 (15.4)	5.22 (4.25‐6.42)		212 (44.4)	143 (56.7)	0.58 (0.41‐0.82)	
N_sx_ MC^c^^,^^d^	4.01 (3.21)	2.55 (2.96)	1.15 (1.12‐1.19)		4.24 (3.28)	3.58 (3.02)	1.08 (1.02‐1.14)	
Sex								
Male	248 (34)	1272 (45)	1 (reference)		149 (31.2)	99 (39.3)	1 (reference)	
Female	481 (66)	1557 (55)	1.57 (1.30‐1.89)		328 (68.8)	153 (60.7)	1.33 (0.94‐1.87)	
Age group (years)								
0‐17	25 (3.4)	229 (8.1)	0.46 (0.25‐0.82)		18 (3.8)	7 (2.8)	1.53 (0.47‐5.03)	
18‐29	70 (9.6)	404 (14.3)	0.63 (0.45‐0.87)		51 (10.7)	19 (7.5)	1.23 (0.64‐2.35)	
30‐39	71 (9.7)	429 (15.2)	0.59 (0.42‐0.81)		47 (9.9)	24 (9.5)	0.99 (0.55‐1.80)	
40‐49	102 (14)	444 (15.7)	0.79 (0.59‐1.06)		79 (16.6)	23 (9.1)	2.00 (1.13‐3.55)	
50‐59	53 (13.8)	220 (15.8)	1 (reference)		132 (27.7)	71 (28.2)	1 (reference)	
60‐69	123 (16.9)	395 (14)	0.79 (0.59‐1.05)		66 (13.8)	57 (22.6)	0.64 (0.39‐1.04)	
70‐79	203 (27.8)	627 (22.2)	0.63 (0.43‐0.91)		40 (8.4)	24 (9.5)	0.97 (0.52‐1.81)	
≥80	71 (9.7)	95 (3.4)	1.16 (0.77‐1.76)		44 (9.2)	27 (10.7)	0.94 (0.49‐1.80)	
Number of pre-existing conditions								
0	297 (40.7)	1563 (55.2)	1 (reference)		196 (41.1)	101 (40.1)	1 (reference)	
1	187 (25.7)	653 (23.1)	1.12 (0.88‐1.42)		113 (23.7)	74 (29.4)	1.10 (0.72‐1.69)	
2	112 (15.4)	328 (11.6)	1.23 (0.92‐1.64)		76 (15.9)	36 (14.3)	1.75 (1.02‐3.00)	
≥3	133 (18.2)	285 (10.1)	1.44 (1.07‐1.96)		92 (19.3)	41 (16.3)	2.01 (1.18‐3.43)	
Weight status according to BMI category								
Normal	327 (44.9)	1435 (50.7)	1 (reference)		221 (46.3)	106 (42.1)	1 (reference)	
Overweight	176 (24.1)	498 (17.6)	1.16 (0.90‐1.50)		113 (23.7)	63 (25)	0.82 (0.55‐1.22)	
Obese	222 (30.5)	884 (31.2)	0.98 (0.79‐1.22)		141 (29.6)	81 (32.1)	0.79 (0.50‐1.23)	
Missing	4 (0.5)	12 (0.4)	0.98 (0.79‐1.22)		2 (0.4)	2 (0.8)	0.16 (0.02‐1.25)	
Education (ISCED^e^)								
Low	80 (11)	356 (12.6)	1.09 (0.78‐1.54)		58 (12.2)	22 (8.7)	1.73 (0.96‐3.15)	
Medium	398 (54.6)	1348 (47.6)	1 (reference)		248 (52)	150 (59.5)	1 (reference)	
High	229 (31.4)	1031 (36.4)	1.19 (0.97‐1.47)		156 (32.7)	73 (29)	1.26 (0.86‐1.84)	
Missing	22 (3)	94 (3.3)	1.95 (1.11‐3.42)		15 (3.1)	7 (2.8)	1.08 (0.39‐2.99)	
Self-reporting								
Self	711 (97.5)	2665 (94.2)	1 (reference)		467 (97.9)	244 (96.8)	1 (reference)	
Proxy	18 (2.5)	164 (5.8)	0.59 (0.33‐1.08)		10 (2.1)	8 (3.2)	0.60 (0.20‐1.80)	

a CAWI: computer-assisted website interviewing.

b CATI: computer-assisted telephone interviewing.

c N_sx_ MC: number of symptoms via multiple choice items.

d Mean (SD).

e ISCED: International Standard Classification of Education.

The answers to free-text items were further investigated by assessing the effect of the mode on whether additional symptoms were identified or extracted at either the time of infection or interview. This was done by conducting a mixed effects logistic regression analysis on the subset of answers from participants who did engage with the free-text items (n=729; right columns in Table 2). The outcomes of the regression analysis showed that the odds of collecting additional symptoms from the free-text items was 42% lower for answers from CATI questionnaires (OR 0.58, 95% CI 0.41‐0.82). These odds rose by 8% for each additional symptom reported in the multiple-choice items (OR 1.08, 95% CI 1.02‐1.14). The odds increased 2-fold for participants who reported 2 or more pre-existing conditions than those reporting none (2: OR 1.8, 95% CI 1.02‐3.00; ≥3: OR 2.0, 95% CI 1.18‐3.43). Participants aged 40 years to 49 years were twice as likely to provide answers that resulted in the collection of additional symptoms (OR 2.0, 95% CI 1.13‐3.55).

**Figure 3.**
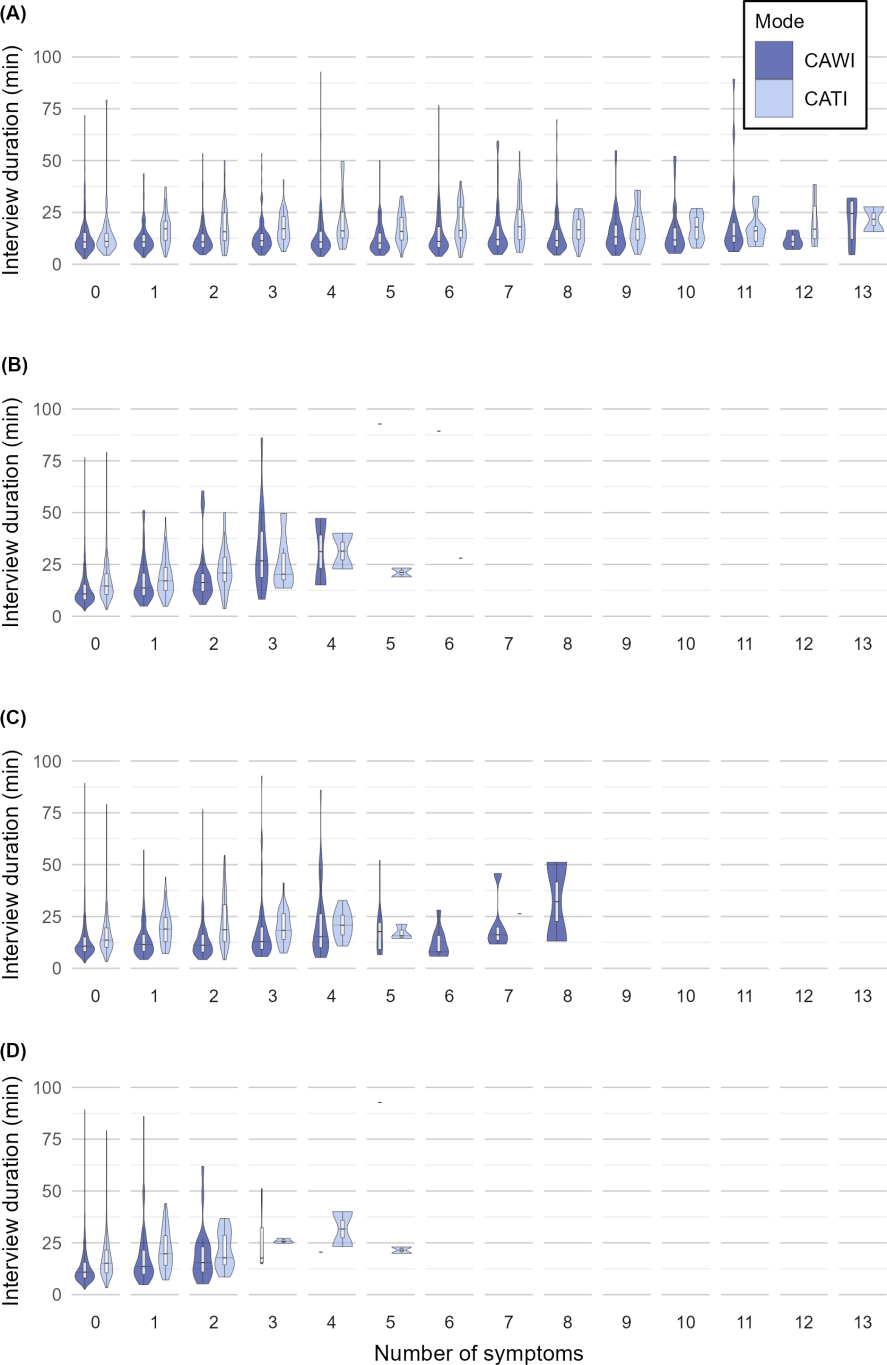
Violin plots with box plot for interview duration (in minutes) of multiple-choice and free-text items on the number of symptoms experienced at the time of infection and interview by interview mode (computer-assisted telephone interviewing [CATI] vs computer-assisted website interviewing [CAWI]): (A) at time of infection in multiple-choice items, (B) at time of infection in free-text items, (C) at time of interview in multiple-choice items, and (D) at time of interview in free-text items.

Some participants’ sociodemographic characteristics, the number of symptoms they reported (Figure 3), and the time they took to complete their interviews were associated with their interview mode preference (Table 3). A gamma generalized linear model with log link function revealed that choosing CATI over CAWI was associated with an 8% increase in interview duration (MR 1.08, 95% CI 1.01‐1.16). Across all types of symptom counts, the duration increased between 2% and 18% per additional symptom reported (infection multiple choice: MR 1.02, 95% CI 1.01‐1.03; infection free-text: MR 1.18, 95% CI 1.13‐1.23; interview multiple choice: MR 1.03, 95% CI 1.01‐1.06; interview free-text: MR 1.09, 95% CI 1.03‐1.15). Reporting a higher number of pre-existing conditions was also associated with longer interview durations (2: MR 1.13, 95% CI 1.03‐1.24; ≥3: MR 1.10, 95% CI 1.00‐1.22). Furthermore, all age groups younger than 50 years had shorter interview durations (0‐17 years: MR 0.84, 95% CI 0.71‐0.99; 18‐29 years: MR 0.80, 95% CI 0.72‐0.88; 30‐39 years: MR 0.80, 95% CI 0.73‐0.88; 40‐49 years: MR 0.84, 95% CI 0.77‐0.92), while participants in the age group of 70 years to 79 years participated in longer interviews (MR 1.26, 95% CI 1.12‐1.42).

**Table 3.** Mean ratios calculated from a gamma generalized linear model with log link function for the outcome interview duration.

Predictors	Interview duration (minutes; n=1779)		Mean ratio (95% CI)	
Mean (SD)	Median (IQR)		
Interview mode				
CAWI^a^	13.9 (9.84)	11.06 (8.06)	1 (reference)	
CATI^b^	18.2 (10.7)	15.90 (11.23)	1.08 (1.01‐1.16)	
N_Sx_^c^ infection MC^d^	—^e^	—	1.02 (1.01‐1.03)	
N_Sx_ infection free-text	—	—	1.18 (1.13‐1.23)	
N_Sx_ interview MC	—	—	1.03 (1.01‐1.06)	
N_Sx_ interview free-text	—	—	1.09 (1.03‐1.15)	
Sex				
Male	15.09 (10.40)	11.76 (9.86)	1 (reference)	
Female	14.74 (10.02)	12.00 (8.78)	0.92 (0.87‐0.98)	
Age group (years)				
0‐17	11.50 (7.16)	9.78 (6.61)	0.84 (0.71‐0.99)	
18‐29	11.91 (7.54)	9.70 (5.77)	0.80 (0.72‐0.88)	
30‐39	12.39 (9.04)	10.18 (6.63)	0.80 (0.73‐0.88)	
40‐49	12.87 (8.03)	10.82 (6.53)	0.84 (0.77‐0.92)	
50‐59	16.27 (11.12)	13.32 (10.35)	1 (reference)	
60‐69	16.88 (10.71)	14.02 (9.29)	1.07 (0.97‐1.17)	
70‐79	20.91 (12.36)	18.75 (13.27)	1.26 (1.12‐1.42)	
≥80	19.80 (11.31)	17.75 (13.82)	1.14 (0.98‐1.33)	
Weight status according to BMI category				
Normal	14.12 (9.06)	11.42 (8.23)	1 (reference)	
Overweight	14.89 (10.24)	12.00 (8.93)	0.95 (0.89‐1.02)	
Obese	16.99 (12.43)	13.57 (11.32)	1.00 (0.92‐1.09)	
Missing	10.33 (3.86)	10.19 (4.03)	0.70 (0.48‐1.09)	
Number of pre-existing conditions				
0	13.17 (8.59)	10.83 (7.29)	1 (reference)	
1	15.09 (10.07)	12.13 (9.11)	1.00 (0.94‐1.08)	
2	17.37 (11.44)	14.41 (10.77)	1.13 (1.03‐1.24)	
≥3	19.49 (13.20)	16.08 (13.77)	1.10 (1.00‐1.22)	
Education (ISCED^f^)				
Low (1, 2)	13.53 (9.49)	10.79 (7.76)	1.00 (0.90‐1.12)	
Medium (3, 4)	15.41 (9.58)	12.80 (9.37)	1 (reference)	
High (5, 6)	14.38 (10.57)	11.09 (9.52)	1.00 (0.94‐1.06)	
Missing	17.57 (15.34)	13.39 (12.56)	1.30 (1.10‐1.55)	
Proxy versus self				
Self	15.03 (10.24)	12.05 (9.33)	1 (reference)	
Proxy	12.17 (8.76)	9.35 (7.84)	0.86 (0.74‐1.01)	

a CAWI: computer-assisted website interviewing.

b CATI: computer-assisted telephone interviewing.

c N_Sx_: number of symptoms.

d MC: multiple choice item.

e Not applicable.

f ISCED: International Standard Classification of Education.

Applying the back-door method [30,31] to disentangle selection and measurement effects revealed a selection effect: The mean number of symptoms collected via the multiple-choice questions on symptoms experienced at the time of infection increased by approximately 0.6 (difference −0.63, 95% CI −0.95 to −0.31) after weighting the CATI sample to align with the CAWI sample (Table 4). When expressed in terms of SMD, this increase corresponded to a small effect size (SMD −0.19, 95% CI−0.33 to −0.05) according to common benchmarks (eg, Cohen *d*: 0.2=small, 0.5=medium, 0.8=large). Noticeable measurement effects were also identified: Symptoms reported via CATI were associated with higher counts collected through multiple-choice items at the time of infection (difference 0.97, 95% CI 0.47 to 1.5), free-text items at the time of infection (difference 0.40, 95% CI 0.29 to 0.52), and free-text items at the time of the interview (difference 0.15, 95% CI 0.05 to 0.25). Quantified as SMD, this amounted to a large effect size for symptom counts collected via free-text items at the time of infection (SMD 0.55, 95% CI 0.43 to 0.66) and medium effects sizes for symptom counts collected via multiple choice at the time of infection (SMD 0.31, 95% CI 0.19 to 0.42) and free text at the time of the interview (SMD 0.25, 95% CI 0.14 to 0.36).

**Table 4.** Selection and measurement effects, quantified as raw differences (D) and standardized mean differences (SMD), in the number of reported symptoms (N_Sx_) collected with either multiple-choice (MC) or free-text items at the times of infection and interview.

N_Sx_	CATI^a^ (n=395), mean (SD)	CATI_W_^b^ (n=395), mean (SD)	CAWI^c^ (n=1384), mean (SD)	Selection (CATI-CATI_W_)		Measurement (CATI_W_-CAWI)	
				D (95% CI)	SMD (95% CI)	D (95% CI)	SMD (95% CI)
Infection MC	5.09 (3.40)	5.72 (3.49)	4.75 (2.93)	−0.63 (−0.95 to −0.31)	−0.19 (−0.33 to −0.05)	0.97 (0.47 to 1.5)	0.31 (0.19 to 0.42)
Infection free-text	0.53 (0.90)	0.55 (0.86)	0.15 (0.54)	−0.02 (−0.10 to 0.06)	−0.02 (−0.16 to 0.12)	0.40 (0.29 to 0.52)	0.55 (0.43 to 0.66)
Interview MC	0.91 (1.26)	0.89 (1.36)	0.86 (1.38)	0.02 (−0.09 to 0.14)	0.02 (−0.12 to 0.16)	0.02 (−0.17 to 0.22)	0.02 (−0.09 to 0.13)
Interview free-text	0.30 (0.75)	0.28 (0.76)	0.13 (0.44)	0.02 (−0.04 to 0.08)	0.03 (−0.11 to 0.17)	0.15 (0.05 to 0.25)	0.25 (0.14 to 0.36)

a CATI: computer-assisted telephone interviewing.

b CATI_w_: set of counterfactual answers created using propensity score weights.

c CAWI: computer-assisted website interviewing.

To investigate whether the overall observed differences in response frequency associated with the interview mode may have influenced the results of the main analysis reported in the original study [27], we repeated its analysis with the additional variables “interview mode” (CATI vs CATI) and “self-reported” (self vs proxy). The comparison between the results of the original and the modified models (Multimedia Appendix 4) revealed minimal differences. Since this analysis excluded all individuals who did not report any symptoms (n=167), we additionally checked for any systematic differences between the latter and participants who reported at least one symptom (n=1612). No evidence for statistically significant differences was found between the distribution of the two subgroups of participants (*χ*²_1_=0.45, *P*=.504; Multimedia Appendix 5).

## Discussion

### Principal Findings

This study examined the effect of using CAWI and CATI on the response patterns of participants in the CoVerlauf study. In particular, the study compared sample characteristics of participants selecting each mode, as well as their responses to multiple-choice and free-text items about their infection and postinfection symptoms.

In total, the number of questionnaires completed by CATI was roughly 3.5 times lower than that completed by CAWI [3]. Although CAWI participants were over 2 times more likely to belong to the highest ISCED levels and tended to be younger (except for minors), the majority of CATI participants were from lower ISCED levels and aged 60 years and older. Participants reporting a higher number of symptoms in the multiple-choice items and who submitted additional post-COVID-19 symptoms as free-text items were also more likely to have chosen CATI to complete the questionnaire. Similar to earlier studies [5-8,36], the self-selection of CoVerlauf participants to either mode was associated with their personal circumstances (eg, age and educational background). In this sense, the possibility of using different modes might have been both a matter of accessibility and inclusion (eg, for individuals with lower digital dexterity) and an opportunity to cater to the needs of individuals with severe postinfection symptoms or unresolved questions about their COVID-19 experience.

The clustering around the mode may also be reflective of a preference to speak directly with the researchers and specialists, as the spoken interaction would potentially enable participants to spontaneously inquire about unresolved questions. Notably, respondents experiencing a higher number of symptoms were more likely to have chosen CATI. Correspondingly, this reasoning further explains why proxy respondents for participants in the lowest age group (eg, concerned parents) could have chosen CATI rather than to self-administer the questionnaire online. However, it must be noted that participants were not given any indication prior to their decision with whom they would be speaking; in this case, the interviews were conducted by study nurses.

Furthermore, the study identified that the use of the two modes may have influenced data collected and resulted in measurement effects, indicating that response behaviors differed across the modes. The odds of answering the free-text items were 5 times higher for CATI respondents, whereas the odds of actually collecting additional symptoms from these questions were only half for respondents of CATI than of CAWI. These findings were also directly reflected in the overall response duration (measured in minutes) of the questionnaires between the two mode groups, where CATI respondents took longer on average to complete both item formats.

A further analysis using the back-door method [30,31] to untwine selection and measurement effects revealed evidence for selection effects only for multiple-choice items inquiring on the number of symptoms at the time of the infection. At the same time, evidence for measurement effects was observed for symptoms collected from multiple-choice items for both time points, as well as for symptoms from free-text items at the time of infection. These patterns suggest that, although participants with more symptoms were more likely to choose CATI over CAWI (ie, selection effect), CATI was also more effective at eliciting a higher number of symptom reports.

These results possibly hint at a “chattiness factor” in spoken interactions, as the use of CATI led to collecting more free-text items although not forcibly leading to identifying any additional symptoms. Survey research argues that direct interaction between interviewer and respondents, such as in the case of CATI, can cause deviations from the standardized interview procedures, even when strict standardized guidelines are used (eg, verbatim reading of questionnaire items, nondirective probing, and the use of neutral feedback) [37,38]. As a result, lengthier and unscripted content may not be fully avoided despite standardization efforts. Moreover, as suggested for health survey research, in atypical situations, such as those experienced by the CoVerlauf respondents, response accuracy, particularly for free-text items, may be associated with the respondent’s ability to seek or receive further clarification rather than on the strict adherence to a standardized protocol [39]. In this sense, the higher number of free-text responses from CATI questionnaires may stem from the immediate and personal interaction rather than from the questions alone. Allegedly, it could also be argued that CAWI is not as effective as CATI at capturing additional information through the free-text items. As highlighted by the analysis on response duration, the difference in time between the two modes for multiple-choice items was proportionally smaller than that of the free-text items, which might be indicative of the lower effectiveness of the online format at engaging respondents to elaborate on further symptoms (eg, due to the effort needed to structure answers in written form rather than express them spontaneously).

Additionally, the study revealed that CoVerlauf participants reporting more symptoms in the multiple-choice items, with ≥2 pre-existing conditions, and aged 40 years to 49 years were the most likely to submit free-text items with identifiable symptoms. Together, the higher numbers of symptoms and pre-existing conditions might be indicative of an increased self-awareness and previous experience describing health conditions to researchers and other health care professionals. Although this was the case for all participants, independently of their interview mode, submitting free-text items may have been particularly pronounced in the CATI interviews by diagnostic biases, occurring when interviewers inadvertently make assumptions about participants’ health status based on answers provided to initial questions in an interview. As the odds of identifying additional symptoms in the free-text items rose with the number of previously stated symptoms in the multiple-choice list, it cannot be ruled out that interviewers varied their intensity or form of asking or probing for follow-up information to the free-text questions. Typically, this effect can be countered by appropriate interviewer training, as was done in the CoVerlauf study. Definitely ruling out a diagnostic bias post hoc, however, would require audio recordings of the telephone interviews, which were not created due to privacy concerns.

Overall, this study found that measurement effects may arise when using a concurrent mixed mode approach. By including the interview mode as a covariate in the original analysis reported in [27], which investigated post-COVID-19 conditions, this study found that the mode effects observed here would not have changed the original conclusions. A generally discussed solution to isolate or mitigate these effects would be to randomly assign eligible participants to available modes and, as such, control for selection biases. However, a randomized single-mode approach would have the drawback of increasing the odds of study withdrawal for participants who find the assigned interview mode inaccessible or unsuitable for sharing their personal information. Such approach of mode randomization would therefore defeat the purpose of inclusion and broadening participation or response.

In this sense, enabling a speech-based response within the CAWI questionnaires, namely for free-text items, could facilitate isolating the mode effects without defeating the purpose of representativeness. Providing such an option could allow researchers to simultaneously isolate the effect of the interviewer’s presence on eliciting open-ended answers and the effort required for participants to type elaborate responses with, for example, support of speech-to-text technology.

### Strengths and Limitations

This analysis made use of a large socioeconomically diverse sample and a combination of different analytical methodologies to explore the problem of mode effects from multiple perspectives.

However, only self-reported data on symptoms and pre-existing conditions were available. Therefore, it was not possible to validate whether the observed differences between CATI and CAWI corresponded to differences in health status (ie, a selection effect). None of the analyses included the severity of symptoms, which may have also influenced the choice of interview mode, because neither question format assessed the severity of the symptoms [27]. The analyses could not control for possible differences in interviewer involvement during the telephone interviews because the interviews were not recorded. Since the interview mode was not randomly assigned, the model adjusting for mode choice might still provide biased estimates (eg, if the mode selection is a result of the outcome or confounds with other unmeasured covariates). Finally, the validity of the back-door method used here critically depends on the assumption that the selected back-door variables fully capture the selection effect on the variables of interest, an assumption that eludes empirical testing. As already highlighted by other studies, current methodologies do not allow for easily detangling self-selection mode effects from measurement mode effects and its factors [40,41].

### Conclusions

Although the choice of interview mode was associated with the frequency of reported symptoms and the number of free-text answers, no evidence was found that the interview mode systematically affected the results or conclusion reported in the CoVerlauf study [27]. Moreover, the use of concurrent elective modes for data collection was effective at covering and engaging participants from different age, education, and health groups in the CoVerlauf study. Concurrent mixed mode approaches to data collection can be an effective solution to address the diversity of participants’ communication needs and preferences without biasing the final results of a population-based study.

## Supplementary material

10.2196/80631 Multimedia Appendix 1 Mean ratios calculated from a gamma generalized linear model with log link function for the outcome interview duration excluding outlier values (n=4).

10.2196/80631 Multimedia Appendix 2 Equations 1, 2, 3, and 4.

10.2196/80631 Multimedia Appendix 3 Distribution of self-report questionnaires by age group across interview modes.

10.2196/80631 Multimedia Appendix 4 Comparison between the results of the original and the modified model including “interview mode” (computer-assisted telephone interviewing [CATI] vs computer-assisted telephone interviewing [CATI]) and “self-reported” (“self” vs “proxy”).

10.2196/80631 Multimedia Appendix 5 Distribution of participants who reported at least one post-COVID-19 symptom and who did not report any across interview modes (n=1779).

## Abbreviations

CATI: computer-assisted telephone interviewing
CAWI: computer-assisted website interviewing
ISCED: International Standard Classification of Education
MICE: multivariate imputation by chained equations
MR: mean ratio
OR: odds ratios
SMD: standardized mean difference
