# Supplementary material for: Mode Effects Between Telephone and Web Interviews in the Post-COVID-19 Questionnaire Survey CoVerlauf: Exploratory Study
Source: JMIR Hum Factors. 2026 Mar 6;13:e80631. doi: 10.2196/80631 (PMC12978930; doi:10.2196/80631)
Supplement: Multimedia Appendix 1 [file humanfactors-v13-e80631-s001.pdf]

Mode effects between telephone and web interview in the post-COVID-19 questionnaire survey CoVerlauf: exploratory study

Paula S. Herrera-Espejel[1,2], Hermann Pohlabeln[3], Lisa Kühne[4], and Stefan Rach[1,2*]

[1] Leibniz Institute for Prevention Research and Epidemiology - BIPS, Department of Epidemiological Methods and Etiological Research, Bremen, Germany.

[2] Leibniz ScienceCampus Digital Public Health, Bremen, Germany.

[3] Leibniz Institute for Prevention Research and Epidemiology - BIPS, Department of Biometry and Data Management, Bremen, Germany.

[4] Faculty of Human and Health Sciences, University of Bremen, Bremen, Germany.

*Correspondence to:

Dr. Stefan Rach

Leibniz Institute for Prevention Research and Epidemiology - BIPS

Achterstr. 30, 28359 Bremen, Germany

rach@leibniz-bips.de, sec-epi@leibniz-bips.de

Multimedia Appendix 1.

Mean ratios calculated from a gamma generalized linear model with log link function for the outcome interview duration excluding outlier values (n=4)

	**Interview Duration (n=1774)**			
Predictors	Mean (SD)	Median (IQR)	Exp(log link)[1] exp(β)	95% Confidence Intervals (CIs)
Interview Mode				
CAWI	13.9 (9.84)	11.06 (8.06)	1	Reference
CATI	18.2 (10.7)	15.90 (11.23)	**1.08**	**1.01 – 1.16**
N_{Sx}[a] Infection MC[b]	-	-	1.02	1.01 – 1.03
N_{Sx} Infection Free[c]	-	-	1.18	1.13 – 1.23
N_{Sx} Interview MC	-	-	1.03	1.01 – 1.05
N_{Sx} Interview Free-Text	-	-	1.08	1.02 – 1.15
Sex				
Male	15.09 (10.40)	11.76 (9.86)	1	Reference
Female	14.74 (10.02)	12.00 (8.78)	0.92	0.87 – 0.98

Age Group				
0-17	11.50 (7.16)	9.78 (6.61)	0.84	0.71 – 0.99
18-29	11.91 (7.54)	9.70 (5.77)	0.80	0.72 – 0.88
30-39	12.39 (9.04)	10.18 (6.63)	0.81	0.73 – 0.89
40-49	12.87 (8.03)	10.82 (6.53)	0.84	0.77 – 0.92
50-59	16.27 (11.12)	13.32 (10.35)	1	Reference
60-69	16.88 (10.71)	14.02 (9.29)	1.07	0.98 – 1.18
70-79	20.91 (12.36)	18.75 (13.27)	1.27	1.12 – 1.43
Over 80	19.80 (11.31)	17.75 (13.82)	1.15	0.99 – 1.33
Weight status (BMI, kg/m²)				
Normal (<25.0)	14.12 (9.06)	11.42 (8.23)	1	Reference
Overweight (25.0–30.0)	14.89 (10.24)	12.00 (8.93)	0.95	0.89 – 1.02
Obese (30.0+)	16.99 (12.43)	13.57 (11.32)	1.00	0.92 – 1.08
Missing	10.33 (3.86)	10.19 (4.03)	0.70	0.48 – 1.09
Number of pre-existing conditions				
0	13.17 (8.59)	10.83 (7.29)	1	Reference
1	15.09 (10.07)	12.13 (9.11)	1.00	0.94 – 1.08
2	17.37 (11.44)	14.41 (10.77)	1.13	1.03 – 1.24
3+	19.49 (13.20)	16.08 (13.77)	1.10	1.00 – 1.22
Education (ISCED)				
Low (1, 2)	13.53 (9.49)	10.79 (7.76)	1.00	0.90 – 1.12
Medium (3, 4)	15.41 (9.58)	12.80 (9.37)	1	Reference
High (5, 6)	14.38 (10.57)	11.09 (9.52)	1.00	0.94 – 1.06
Missing	17.57 (15.34)	13.39 (12.56)	1.30	1.10 – 1.55
Proxy vs Self				
Self	15.03 (10.24)	12.05 (9.33)	1	Reference
Proxy	12.17 (8.76)	9.35 (7.84)	0.86	0.74 – 1.00

[a] Number of symptoms, [b] Multiple choice item, [c] Free-text item
