# Supplementary material for: Mode Effects Between Telephone and Web Interviews in the Post-COVID-19 Questionnaire Survey CoVerlauf: Exploratory Study
Source: JMIR Hum Factors. 2026 Mar 6;13:e80631. doi: 10.2196/80631 (PMC12978930; doi:10.2196/80631)
Supplement: Multimedia Appendix 2 [file humanfactors-v13-e80631-s002.pdf]

Mode effects between telephone and web interview in the post-COVID-19 questionnaire survey CoVerlauf: exploratory study

Paula S. Herrera-Espejel[1,2], Hermann Pohlabeln[3], Lisa Kühne[4], and Stefan Rach[1,2*]

[1] Leibniz Institute for Prevention Research and Epidemiology - BIPS, Department of Epidemiological Methods and Etiological Research, Bremen, Germany.
[2] Leibniz ScienceCampus Digital Public Health, Bremen, Germany.
[3] Leibniz Institute for Prevention Research and Epidemiology - BIPS, Department of Biometry and Data Management, Bremen, Germany.
[4] Faculty of Human and Health Sciences, University of Bremen, Bremen, Germany.

*Correspondence to:

Dr. Stefan Rach

Leibniz Institute for Prevention Research and Epidemiology - BIPS

Achterstr. 30, 28359 Bremen, Germany

rach@leibniz-bips.de, sec-epi@leibniz-bips.de

Multimedia Appendix 2. Equations

Equation 1. Multiple logistic regression model for probability of selecting CAWI (online questionnaire) vs. CATI (telephone interview)

$$logit(P(mode)) = ln\left(\frac{P(Y=1)}{1-P(Y=1)}\right)$$
$$= \beta_0 + \beta_1(N_{MC_1}) + \beta_2(N_{FT_1}) + \beta_3(N_{MC_2}) + \beta_4(N_{FT_2}) + \beta_5(Sex) + \beta_6(AgeGroup) + \beta_7(WeightStatus) + \beta_8(N_{PRE-COND}) + \beta_9(Education) + \beta_{10}(Proxy) + \varepsilon$$

where:
mode = 1 for CAWI questionnaires; 0 for CATI questionnaires,
NMC_1 = Number of items from multiple-choice items at infection (i.e., number of symptoms collected from multiple-choice question asking on experienced symptoms at the time of infection),
NMC2 = Number of items from multiple-choice items at interview (i.e., number of symptoms collected from multiple-choice question asking on experienced symptoms at the time of interview),
NFT1 = Number of items from free-text items at infection (i.e., number of symptoms collected from multiple-choice question asking on experienced symptoms at the time of infection),
NFT2 = Number of items from free-text items at interview (i.e., number of symptoms collected from multiple-choice question asking on experienced symptoms at the time of interview),
NMC1 = Number of items from multiple-choice items at infection (i.e., number of symptoms collected from multiple-choice question asking on experienced symptoms at the time of infection),
Sex = 1 for female respondents; 0 for male respondents (reference category),
Age Group is a categorical variable with eight intervals with reference category "50-59" and as indicated above (0-17, 18-29, 30-39, 40-49, 50-59, 60-69, 70-79, and Over 80),
NPRE-COND = Number of pre-existing conditions as reported by respondent,
ISCED is a categorical variable with three groups: low (levels 1 & 2, reference), medium (level 3), high (levels 5 & 6), and
Proxy = 1 if the questionnaire was answered by a proxy respondent; 0 if answered by the participant themselves ("self-response).

Equation 2. Mixed-effects logistic regression model for probability of answering free-text items ("response" vs. "no response")

$$logit(P(freetext)) = ln\left(\frac{P(Y=1)}{1-P(Y=1)}\right)$$
$$= \beta_0 + \beta_1(mode) + \beta_2(N_{MC_1,2}) + \beta_3(Sex) + \beta 4(AgeGroup) + \beta_5(WeightStatus) + \beta 6(N_{PRE-COND}) + \beta_7(Education) + \beta_8(Proxy) + \varepsilon$$

where:

freetext = 1 for free-text items answered by the respondents, 0 for non-responses; and

mode, $N_{MC1,2,}$ Sex, Age Group, ISCED, and Proxy are defined as in Equation 1.

Equation 3. Mixed-effects logistic regression model for probability collecting at least one or more additional symptoms from the free-text items

$$logit(P(\geq 1items)) = ln\left(\frac{P(Y=1)}{1-P(Y=1)}\right)$$
$$= \beta_0 + \beta_1(mode) + \beta_2(N_{MC_1,2}) + \beta_3(Sex) + \beta 4(AgeGroup) + \beta_5(WeightStatus) + \beta 6(N_{PRE-COND}) + \beta_7(Education) + \beta_8(Proxy) + \varepsilon$$

where:

≥1items = 1 for respondents of free-text items which provided at least one symptom, 0 for those who provided none; and

mode, $N_{MC1,2,}$ Sex, Age Group, ISCED, and Proxy are defined as in Equation 2.

Equation 4. Gamma generalized linear regression model with log link function for response duration of questionnaire

$$log(\mu_i) = log\left(E[Yi \mid xi]\right)$$
$$= \beta_0 + \beta_1(mode) + \beta_2(N_{MC_1}) + \beta_3(N_{FT_1}) + \beta_4(N_{MC_2}) + \beta_5(N_{FT_2}) + \beta_6(Sex) + \beta_7(AgeGroup) + \beta_8(WeightStatus) + \beta_9(N_{PRE-COND}) + \beta_{10}(Education) + \beta_{11}(Proxy) + \varepsilon$$

where:

Yi denotes the response duration (minutes) for each respondent, x_i, and

mode, $N_{MC1,2,}$ Sex, Age Group, ISCED, and Proxy are defined as in Equation 1.
