# Supplementary material for: Mode Effects Between Telephone and Web Interviews in the Post-COVID-19 Questionnaire Survey CoVerlauf: Exploratory Study
Source: JMIR Hum Factors. 2026 Mar 6;13:e80631. doi: 10.2196/80631 (PMC12978930; doi:10.2196/80631)
Supplement: Multimedia Appendix 3 [file humanfactors-v13-e80631-s003.pdf]

Mode effects between telephone and web interview in the post-COVID-19 questionnaire survey CoVerlauf: exploratory study

Paula S. Herrera-Espejel[1,2], Hermann Pohlabeln[3], Lisa Kühne[4], and Stefan Rach[1,2*]

[1] Leibniz Institute for Prevention Research and Epidemiology - BIPS, Department of Epidemiological Methods and Etiological Research, Bremen, Germany.
[2] Leibniz ScienceCampus Digital Public Health, Bremen, Germany.
[3] Leibniz Institute for Prevention Research and Epidemiology - BIPS, Department of Biometry and Data Management, Bremen, Germany.
[4] Faculty of Human and Health Sciences, University of Bremen, Bremen, Germany.

*Correspondence to:

Dr. Stefan Rach

Leibniz Institute for Prevention Research and Epidemiology - BIPS

Achterstr. 30, 28359 Bremen, Germany

rach@leibniz-bips.de

Multimedia Appendix 3. Distribution of self-report questionnaires by age group across interview modes.

Respondent:	Self-Reporting (N= 1688)		Proxy Reporting (N=91)	
Interview Mode:	CAWI (N=1307)	CATI (N=381)	CAWI (N=77)	CATI (N=14)
Age group:				
[0-17]	49 (3.7%)	17 (4.5%)	53 (68.8%)	8 (57.1%)
[18-29]	211 (16.1%)	26 (6.8%)	0 (0%)	0 (0%)
[30-39]	219 (16.8%)	28 (7.3%)	3 (3.9%)	0 (0%)
[40-49]	228 (17.4%)	39 (10.2%)	6 (7.8%)	0 (0%)
[50-59]	339 (25.9%)	76 (19.9%)	0 (0%)	0 (0%)
[60-69]	178 (13.6%)	78 (20.5%)	3 (3.9%)	0 (0%)
[70-79]	68 (5.2%)	61 (16.0%)	5 (6.5%)	1 (7.1%)
[80+]	15 (1.1%)	56 (14.7%)	7 (9.1%)	5 (35.7%)
