# Supplementary material for: Mode Effects Between Telephone and Web Interviews in the Post-COVID-19 Questionnaire Survey CoVerlauf: Exploratory Study
Source: JMIR Hum Factors. 2026 Mar 6;13:e80631. doi: 10.2196/80631 (PMC12978930; doi:10.2196/80631)
Supplement: Multimedia Appendix 4 [file humanfactors-v13-e80631-s004.pdf]

Mode effects between telephone and web interview in the post-COVID-19 questionnaire survey CoVerlauf: exploratory study

Paula S. Herrera-Espejel[1,2], Hermann Pohlabeln[3], Lisa Kühne[4], and Stefan Rach[1,2*]

[1] Leibniz Institute for Prevention Research and Epidemiology - BIPS, Department of Epidemiological Methods and Etiological Research, Bremen, Germany.
[2] Leibniz ScienceCampus Digital Public Health, Bremen, Germany.
[3] Leibniz Institute for Prevention Research and Epidemiology - BIPS, Department of Biometry and Data Management, Bremen, Germany.
[4] Faculty of Human and Health Sciences, University of Bremen, Bremen, Germany.

*Correspondence to:

Dr. Stefan Rach

Leibniz Institute for Prevention Research and Epidemiology - BIPS

Achterstr. 30, 28359 Bremen, Germany

rach@leibniz-bips.de

Multimedia Appendix 4. Comparison between the results of the original and the modified model including "interview mode" (CATI vs. CATI) and "self-reported" ("self" vs "proxy")

	Post-COVID-19 condition							
	No		Yes		Rach et al. 2023		Current analysis	
	n	%	n	%	OR	95% CI	OR	95% CI
**Sex**								
Male	611	91,3	58	8,7	Ref.		Ref.	
Female	798	84,6	145	15,4	1,54	(1,05; 2,24)	1,54	(1,06; 2,25)
**Age (mean, sd)**	46,6	18,9	51,7	15,1	1,00	(0,99; 1,01)	1,00	(0,99; 1,02)
**Fatigue**								
No	417	95,9	18	4,1	Ref.		Ref.	
Yes	992	84,3	185	15,7	1,75	(1,00; 3,06)	1,76	(1,01; 3,07)
**Breathing difficulties**								
No	1051	94,3	63	5,7	Ref.		Ref.	
Yes	358	71,9	140	28,1	4,02	(2,80; 5,77)	4,03	(2,81; 5,79)
**Cognitive symptoms**								
No	1377	88,4	181	11,6	Ref.		Ref.	
Yes	32	59,3	22	40,7	2,98	(1,48; 6,02)	3,05	(1,50; 6,18)
**Digestive symptoms**								
No	1098	90,2	119	9,8	Ref.		Ref.	
Yes	311	78,7	84	21,3	1,24	(0,86; 1,80)	1,27	(0,87; 1,84)
**Fever**								
No	665	89,6	77	10,4	Ref.		Ref.	
Yes	744	85,5	126	14,5	0,87	(0,61; 1,25)	0,87	(0,61; 1,25)
**Head-& Boneaches**								
No	454	94,4	27	5,6	Ref.		Ref.	

Yes	955	84,4	176	15,6	2,06	(1,25; 3,42)	2,05	(1,24; 3,38)
**Other respiratory symptoms**								
No	451	90,9	45	9,1	Ref.		Ref.	
Yes	958	85,8	158	14,2	1,16	(0,76; 1,76)	1,16	(0,77; 1,77)
**Changes to sense of smell and taste**								
No	609	89,0	75	11,0	Ref.		Ref.	
Yes	800	86,2	128	13,8	0,91	(0,64; 1,31)	0,91	(0,64; 1,31)
**Heart & circulatory problems**								
No	1375	88,1	186	11,9	Ref.		Ref.	
Yes	34	66,7	17	33,3	1,51	(0,71; 3,24)	1,53	(0,71; 3,29)
**Other Covid-19-related symptoms**								
No	1340	87,9	184	12,1	Ref.		Ref.	
Yes	69	78,4	19	21,6	0,84	(0,44; 1,61)	0,84	(0,44; 1,63)
**Other unspecific symptoms**								
No	1314	88,3	174	11,7	Ref.		Ref.	
Yes	95	76,6	29	23,4	1,37	(0,78; 2,39)	1,43	(0,81; 2,54)
**Hypertension**								
No	1041	88,6	134	11,4	Ref.		Ref.	
Yes	368	84,2	69	15,8	0,87	(0,58; 1,31)	0,88	(0,58; 1,34)
**Other cardiovascular diseases**[a]								
No	1307	88,0	179	12,0	Ref.		Ref.	
Yes	102	81,0	24	19,0	0,80	(0,43; 1,50)	0,81	(0,44; 1,52)
**Diabetes mellitus**								
No	1335	87,7	187	12,3	Ref.		Ref.	
Yes	74	82,2	16	17,8	0,72	(0,36; 1,44)	0,71	(0,35; 1,43)
**Asthma**								
No	1268	88,3	168	11,7	Ref.		Ref.	
Yes	141	80,1	35	19,9	1,17	(0,67; 2,05)	1,14	(0,65; 2,01)
**Chronic lung disease (e.g., COPD)**								
No	1302	88,6	168	11,4	Ref.		Ref.	
Yes	107	75,4	35	24,6	1,49	(0,83; 2,66)	1,52	(0,85; 2,73)
**Chronic bronchitis**								
No	1345	88,4	177	11,6	Ref.		Ref.	
Yes	64	71,1	26	28,9	1,30	(0,71; 2,38)	1,28	(0,70; 2,34)
**Cancer**								
No	1323	88,0	180	12,0	Ref.		Ref.	
Yes	86	78,9	23	21,1	1,65	(0,79; 3,48)	1,68	(0,80; 3,53)
**Currently receiving medical treatment for cancer**								
No	1377	87,8	192	12,2	Ref.		Ref.	
Yes	32	74,4	11	25,6	0,83	(0,28; 2,51)	0,83	(0,28; 2,52)
**Weakened immune system**[b]								
No	1322	88,5	172	11,5	Ref.		Ref.	
Yes	87	73,7	31	26,3	1,57	(0,92; 2,69)	1,58	(0,93; 2,71)
**Chronic liver disease**								
No	1385	87,6	196	12,4	Ref.		Ref.	
Yes	24	77,4	7	22,6	0,99	(0,34; 2,90)	1,02	(0,35; 3,01)
**Adiposity (diagnosed by physician)**								
No	1313	88,1	177	11,9	Ref.		Ref.	
Yes	96	78,7	26	21,3	1,06	(0,56; 2,01)	1,03	(0,54; 1,95)
**Education (ISCED)**								
Low (ISCED 1,2)	161	87,5	23	12,5	1,39	(0,74; 2,58)	1,44	(0,76; 2,74)
Medium (ISCED 3,4)	683	85,2	119	14,8	1,24	(0,84; 1,84)	1,27	(0,85; 1,88)
High (ISCED 5,6)	524	90,2	57	9,8	Ref.		Ref.	
Missing	41	91,1	4	8,9	1,04	(0,28; 3,90)	1,00	(0,25; 3,95)

**Obese (BMI: >30)**[c]								
No	1164	89,1	142	10,9	Ref.		Ref.	
Yes	245	80,1	61	19,9	1,53	(0,97; 2,41)	1,55	(0,98; 2,46)
**Smoking status**								
Never smoked	837	89,4	99	10,6	Ref.		Ref.	
Current or ex-smoker	572	84,6	104	15,4	1,19	(0,84; 1,69)	1,19	(0,83; 1,69)
**Physician consulted**								
No	1121	90,3	120	9,7	Ref.		Ref.	
Yes	288	77,6	83	22,4	2,25	(1,54; 3,30)	2,14	(1,44; 3,18)
**Hospitalized**								
No	1334	89,1	164	10,9	Ref.		Ref.	
Yes	75	65,8	39	34,2	3,68	(2,08; 6,49)	3,72	(2,11; 6,58)
**Time interval infection to interview**								
90-120 days	481	85,4	82	14,6	Ref.		Ref.	
120-180 days	425	87,8	59	12,2	0,71	(0,47; 1,08)	0,72	(0,47; 1,10)
>180 days	503	89,0	62	11,0	0,63	(0,42; 0,95)	0,64	(0,43; 0,96)
**Mode**								
CAWI	1105	78,4	153	75,4			Ref.	
CATI	304	21,6	50	24,6			0,80	(0,50; 1,27)
**Self-filled**								
No	62	4,4	6	3,0			Ref.	
Yes	1347	95,6	197	97,0			1,06	(0,39; 2,91)
**All**	1409	87,4	203	12,6				

[a] Includes circulatory problems of the heart, stenosis of the coronary arteries, angina pectoris, heart attack, heart failure or heart insufficiency, stroke

[b] Medical conditions associated with weakened immune system or under medications that weaken the immune system

[c] Calculated from weight and height as reported in questionnaire
