# Supplementary material for: Mode Effects Between Telephone and Web Interviews in the Post-COVID-19 Questionnaire Survey CoVerlauf: Exploratory Study
Source: JMIR Hum Factors. 2026 Mar 6;13:e80631. doi: 10.2196/80631 (PMC12978930; doi:10.2196/80631)
Supplement: Multimedia Appendix 5 [file humanfactors-v13-e80631-s005.pdf]

Mode effects between telephone and web interview in the post-COVID-19 questionnaire survey CoVerlauf: exploratory study

Paula S. Herrera-Espejel[1,2], Hermann Pohlabeln[3], Lisa Kühne[4], and Stefan Rach[1,2*]

[1] Leibniz Institute for Prevention Research and Epidemiology - BIPS, Department of Epidemiological Methods and Etiological Research, Bremen, Germany.
[2] Leibniz ScienceCampus Digital Public Health, Bremen, Germany.
[3] Leibniz Institute for Prevention Research and Epidemiology - BIPS, Department of Biometry and Data Management, Bremen, Germany.
[4] Faculty of Human and Health Sciences, University of Bremen, Bremen, Germany.

*Correspondence to:

Dr. Stefan Rach

Leibniz Institute for Prevention Research and Epidemiology - BIPS

Achterstr. 30, 28359 Bremen, Germany

rach@leibniz-bips.de, sec-epi@leibniz-bips.de

Multimedia Appendix 5. Distribution of participants who reported at least one post-COVID-19 symptoms and who did not report any across interview modes (n=1779).

**Overall Participants (n=1779)**	**At least one symptom in either or both multiple-choice and free-text items (n=1612)**		**No symptoms in either question format (n=167)**	
Interview Mode	N	%	N	%
CAWI (n=1384)	1258	78.0%	126	75.5%
CATI (n=395)	354	22.0%	41	24.5%

Pearson's Chi-squared test with Yates' continuity correction: X^2 (1) = 0.44752, p-value = 0.5035
